# Optical Coherence Tomography Evaluation of Peripapillary and Macular Structure Changes in Pre-perimetric Glaucoma, Early Perimetric Glaucoma, and Ocular Hypertension: A Systematic Review and Meta-Analysis

**DOI:** 10.3389/fmed.2021.696004

**Published:** 2021-07-01

**Authors:** Yuxin Tong, Tiantian Wang, Xinyu Zhang, Yi He, Bing Jiang

**Affiliations:** ^1^Department of Ophthalmology, Second Xiangya Hospital, Central South University, Changsha, China; ^2^Hunan Clinical Research Center of Ophthalmic Disease, Changsha, China; ^3^Department of Ophthalmology, Xiangya Hospital, Central South University, Changsha, China; ^4^Department of Neurosurgery, Xiangya Hospital, Central South University, Changsha, China

**Keywords:** pre-perimetric glaucoma, early perimetric glaucoma, ocular hypertension, optical coherence tomography, retinal nerve fiber layer, ganglion cell plus inner plexiform layer, ganglion cell complex

## Abstract

**Background:** This study aimed to assess the differences in the average and sectoral peripapillary retinal nerve fiber layer (pRNFL), macular ganglion cell plus inner plexiform layer (mGCIPL), and macular ganglion cell complex (mGCC) thickness using optical coherence tomography (OCT) in patients with pre-perimetric glaucoma (PPG) compared to those with early perimetric glaucoma (EG) and ocular hypertension (OHT).

**Methods:** A comprehensive literature search of the PubMed database, the Cochrane Library, and Embase was performed from inception to March 2021. The weighted mean difference (WMD) with the 95% confidence interval (CI) was pooled for continuous outcomes.

**Results:** Twenty-three cross-sectional studies comprising 2,574 eyes (1,101 PPG eyes, 1,233 EG eyes, and 240 OHT eyes) were included in the systematic review and meta-analysis. The pooled results demonstrated that the average pRNFL (WMD = 8.22, 95% CI = 6.32–10.12, *P* < 0.00001), mGCIPL (WMD = 4.83, 95% CI = 3.43–6.23, *P* < 0.00001), and mGCC (WMD = 7.19, 95% CI = 4.52–9.85, *P* < 0.00001) were significantly thinner in patients with EG than in those with PPG. The sectoral thickness of pRNFL, mGCIPL, and mGCC were also significantly lower in the EG eyes. In addition, the average pRNFL and mGCC were significantly thinner in the PPG eyes than those in the OHT eyes (pRNFL: WMD = −8.57, 95% CI = −9.88 to −7.27, *P* < 0.00001; mGCC: WMD = −3.23, 95% CI = −6.03 to −0.44, *P* = 0.02). Similarly, the sectoral pRNFL and mGCC were also significantly thinner in the PPG eyes than those in the OHT eyes.

**Conclusion:** OCT-based measurements of peripapillary and macular structural alterations can be used to distinguish PPG from EG and OHT, which can help understand the pathophysiology of glaucoma at earlier stages. Studies that employ clock hour classification methods and longitudinal studies are needed to verify our findings.

**Systematic Review Registration:** https://www.crd.york.ac.uk/prospero/display_record.php?RecordID=239798 CRD42021239798

## Introduction

Glaucoma is a group of neurodegenerative diseases that is characterized by the progressive loss of retinal ganglion cells (RGCs) and axons, followed by the irreversible visual field (VF) deterioration (1). Glaucoma is one of the leading causes of blindness, and ~111.8 million people worldwide are expected to suffer from glaucomatous optic neuropathy through 2040. This imposes a huge burden on public health systems (2). Elevated intraocular pressure (IOP) is believed to be one of the major risk factors of glaucoma. Patients that have increased IOP with normal appearance of the optic disc can have about nine times the risk of developing glaucoma and are regarded as ocular hypertension (OHT) individuals or glaucoma suspects (3). Currently, reducing the IOP is the only effective method for glaucoma treatment (4, 5). However, since glaucoma has an insidious onset and obscure symptoms especially at the earlier stages, such as pre-perimetric glaucoma (PPG) and early perimetric glaucoma (EG), patients are usually diagnosed at an advanced stage with severe VF defects (2, 6). Thus, early detection of glaucomatous damage is crucial for hypotensive therapies to slow glaucoma progression and ameliorate the quality of life (7, 8).

Several methods have been utilized for the diagnosis of glaucoma, and of these, standard automated perimetry-based VF examination is the gold standard for evaluating the severity of glaucomatous damage (9). Nevertheless, studies have shown that ganglion cell loss can precede VF defects in glaucoma (10–18) indicating that morphological changes occur earlier than functional damage. Since severe functional damage is closely related to the central region of the VF, it is difficult to solely rely on poor patient performance in standard automated perimetry (19). Moreover, the VF test is occasionally unreliable, which impairs its diagnostic efficacy. Therefore, more objective and reproducible methods are required for assessing the peripapillary and macular structure changes in glaucoma.

Optical coherence tomography (OCT) is a quantitative and non-invasive method of enhanced-depth visualization of the optic nerve head (ONH) and retina with high imaging quality and scanning speed, which enables clinicians to monitor morphological changes of the ONH and retina in glaucoma (20–24). Several studies using OCT have reported that the attenuation of the peripapillary retinal nerve fiber layer (pRNFL), macular ganglion cell plus inner plexiform layer (GCIPL), and macular ganglion cell complex (mGCC) could be hallmark features of glaucoma (20, 22, 24, 25). Although some investigations have revealed the continuum of glaucoma from mild to advanced stages, studies have shown inconsistencies regarding the diagnostic values of OCT indicators in differentiating PPG from EG (26–44) and in differentiating PPG from OHT (39, 43, 45–48).

Thus, we conducted this systematic review and meta-analysis to facilitate a better understanding of glaucomatous progression form OHT without apparent ONH configuration changes to the pre-perimetric stage with structural deterioration, and from the pre-perimetric to the early perimetric stage with impaired VF in view of the peripapillary and macular structural alterations, and to enable ophthalmologists to discriminate PPG from EG, and PPG from OHT.

## Methods

This systematic review and meta-analysis adhered to the Preferred Reporting Items for Systematic Reviews and Meta-analysis (PRISMA) statement methodology and the Meta-analysis of Observational Studies in Epidemiology (MOOSE) guidelines (49, 50). Three investigators (YT, TW, and YH) independently performed the literature search, data extraction, and quality assessment based on the same standard. The study was registered in PROSPERO (CRD42021239798).

### Literature Search

We performed a comprehensive literature search of the PubMed database, Embase, and the Cochrane Library from inception to March 2021 using the following strategy with the combination of free text terms and Medical Subject Headings (MeSH): “preperimetric”[All Fields] AND (“glaucoma”[MeSH Terms] OR “glaucoma”[All Fields] OR “glaucomas”[All Fields]) AND (“ocular hypertension”[MeSH Terms] OR (“ocular”[All Fields] AND “hypertension”[All Fields]) OR “ocular hypertension”[All Fields] OR (“suspect”[All Fields] OR “suspected”[All Fields] OR “suspecting”[All Fields] OR “suspects”[All Fields]) OR “early”[All Fields] OR “mild”[All Fields] OR (“hypertense”[All Fields] OR “hypertension”[MeSH Terms] OR “hypertension”[All Fields] OR “hypertension s”[All Fields] OR “hypertensions”[All Fields] OR “hypertensive”[All Fields] OR “hypertensive s”[All Fields] OR “hypertensives”[All Fields])) AND (“tomography, optical coherence”[MeSH Terms] OR (“tomography”[All Fields] AND “optical”[All Fields] AND “coherence”[All Fields]) OR “optical coherence tomography”[All Fields] OR (“optical”[All Fields] AND “coherence”[All Fields] AND “tomography”[All Fields]) OR “OCT”[All Fields]). We modified search strategies according to the different requirements of the different databases. Full-text screening was conducted to include potentially applicable studies.

### Inclusion and Exclusion Criteria

Eligible studies were included if they fulfilled the following criteria: (1) original article; (2) inclusion of PPG and (EG or OHT) with the same diagnostic standard: for PPG, patients had to demonstrate characteristic glaucomatous optic nerve damage (i.e., neuroretinal rim thinning, excavation, or notching) without a reproducible VF; for EG, in addition to the typical glaucomatous optic disc changes (i.e., neuroretinal rim thinning, excavation, or notching), the mean deviation (MD) of the VF defect had to exceed −6 dB based on the Hodapp-Anderson-Parrish VF severity grading scale (51); for OHT, patients with an elevated IOP > 21 mmHg but with normal optic disc appearance and VF were included; and (3) inclusion of at least one of the following quantitative indicators measured by OCT – pRNFL, mGCIPL, or mGCC thickness.

The exclusion criteria were: (1) animal experiments, reviews, case reports, and conference abstracts; (2) non-inclusion of PPG, EG and OHT; (3) lack of information regarding pRNFL, mGCIPL, or mGCC thickness; (4) different diagnostic standards; and (5) studies with unextractable data.

### Data Extraction

The following details were extracted with regard to the studies: title, first author, publication year, study type, region, source of patients, number of patients and eyes, mean age of patients, female/male ratios, type of spectral domain OCT (SD-OCT) or time domain OCT (TD-OCT) devices, type of glaucoma, diagnostic standards, average and sectoral pRNFL, mGCIPL and mGCC thickness, scan area and protocol of the ONH and macular region, and MD of the VF. Disagreements were resolved through consensus after discussion among all authors.

### Quality Assessment

The Agency for Healthcare Research and Quality (AHRQ) methodology checklist was used to evaluate the quality of the included cross-sectional studies.

### Statistical Analyses

All statistical analyses were conducted using Review Manager V5.4.1 (Cochrane Collaboration, London, United Kingdom) and Stata V12.0 (StataCorp LLC, Texas, America). We employed the weighted mean difference (WMD) with the 95% confidence interval (CI) to pool the mean differences of OCT parameters between the PPG and EG groups, and between the PPG and OHT groups. A *P*-value of < 0.05 was considered to be statistically significant. Heterogeneity was estimated using Cochrane's Q test and I^2^ statistics. A fixed-effects model was used when I^2^ < 50% (52); otherwise, a random-effects model (Der Simonian-Laird method) was used. We performed subgroup analyses according to the type of glaucoma, type of OCT device, and macular scan area. Subgroups with less than two included studies were excluded to prevent further discrepancy. “Leave-one-out” sensitivity analyses were performed to validate the stability and reliability of the results. Publication bias was evaluated by the combination of Begg's funnel plot and Egger's test (53, 54).

In some articles, the eight-quadrant classification method was used to display sectoral thickness while other studies employed the four-quadrant classification method. Since most of the studies used the four-quadrant classification (only one study used the eight-quadrant classification, which we included for the combined analysis regarding the pRNFL thickness), we transformed the eight-quadrant data to four-quadrant data to reduce heterogeneity using the following formula to combine the means and standard deviations of the two groups:

$$\begin{array}{l} {\bar{x}}_{12}=\frac{N_{1}\cdot {\bar{x}}_{1}+N_{2}\cdot {\bar{x}}_{2}}{N_{1}+N_{2}}\\ \sigma_{12}=\sqrt{\frac{\left(N_{1}-1\right)\cdot \sigma_{1}^{2}+\left(N_{2}-1\right)\cdot \sigma_{2}^{2}+\frac{N_{1}\cdot N_{2}}{N_{1}+N_{2}}\cdot \left({\bar{x}}_{1}^{2}+{\bar{x}}_{2}^{2}-2{\bar{x}}_{1}\cdot {\bar{x}}_{2}\right)}{N_{1}+N_{2}-1}} \end{array}$$

${\bar{x}}_{1}$ and ${\bar{x}}_{2}$ are the mean pRNFL or mGCIPL or mGCC thickness of the two adjacent sections in the eight-quadrant classification. σ_1_ and σ_2_ are the standard deviations of the two groups. *N*_1_ and *N*_2_ are number of the eyes in the two sections. ${\bar{x}}_{12}$ and σ_12_ are the combined mean and standard deviation (i.e., ${\bar{x}}_{1}$ and σ_1_ refer to parameters of the temporal superior quadrant, ${\bar{x}}_{2}$ and σ_2_ refer to parameters of the temporal inferior quadrant, and ${\bar{x}}_{12}$ and σ_12_ refer to combined parameters of the temporal superior and inferior quadrants).

## Results

### Literature Search

**Figure 1:**
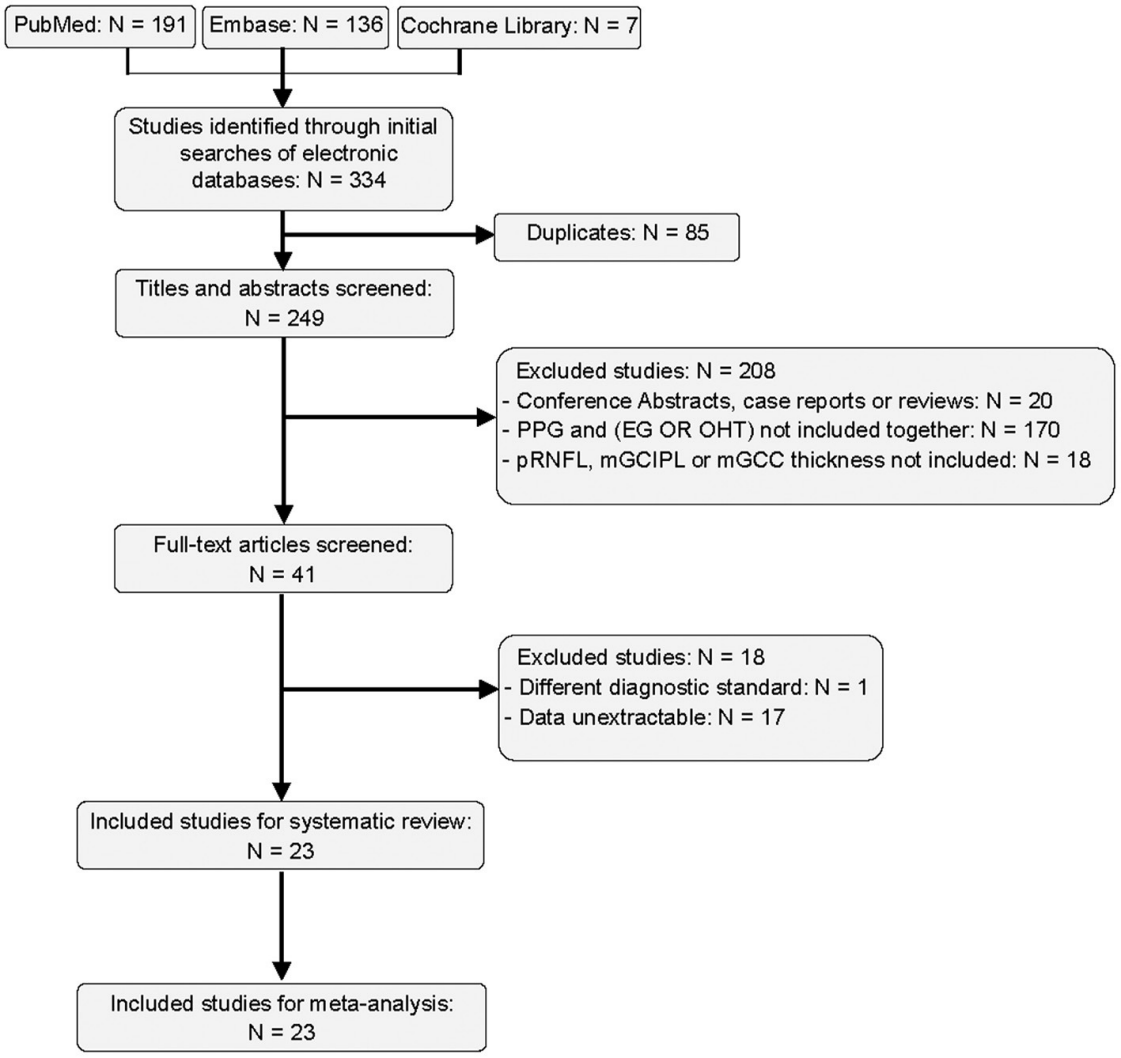
Flow diagram of included studies.

A total of 334 studies were retrieved in our screening, of which 85 duplicates were removed and 208 articles were excluded by titles and abstracts. We further excluded 17 studies with unextractable data and 1 study that used a different diagnostic standard. Finally, 23 studies were integrated into the qualitative and quantitative analyses (26–48). The flow diagram of literature search is shown in Figure 1.

### Characteristics of Included Studies

According to our eligibility criteria, 23 cross-sectional studies comprising 2,574 eyes (1,101 PPG eyes, 1,233 EG eyes, and 240 OHT eyes) were included in the systematic review and the meta-analysis. Detailed characteristics of the included studies are summarized in Table 1. The AHRQ checklist scores of all the included cross-sectional studies were not <5, demonstrating that the studies were of good quality. Details are presented in Table 2.

**Table 1:** Characteristics of included studies.

**Study**	**Year**	**Region**	**No. eyes**			**Mean age** **±** **SD (yrs)**			**Gender (F/M)**			**Device (OCT)**	**Glaucoma types**	**Main outcome**	**Scan area (mm**^****2****^ **or mm**^****3****^**)**	
			**PPG**	**EG**	**OHT**	**PPG**	**EG**	**OHT**	**PPG**	**EG**	**OHT**				**ONH**	**Macular region**
Wang et al. (44)	2020	China	26	22		36.9 ± 11.1	41.4 ± 13.9		6/16	7/12		RTVue	POAG	pRNFL, GCC		6 × 6
Sarigül Sezenöz et al. (43)	2020	Turkey	28	31	18	66.71 ± 11.33	68.20 ± 9.12	66.61 ± 10.44	N/A	N/A	N/A	Heidelberg	Mixed	pRNFL, GCC, total retina thickness, G/T ratio		
Lu et al. (42)	2020	China	44	42		43 ± 11	46 ± 11		17/27	20/22		RTVue	POAG	pRNFL, GCC	4.5 × 4.5	6 × 6
Hou et al. (41)	2019	USA	68	162		68.4 ± 10.8	68.4 ± 8.6		30/25	60/61		RTVue Spectralis	POAG	pRNFL, GCC		3 × 3
Kim et al. (40)	2017	Korea	26	26		52.08 ± 11.77	55.65 ± 13.36		16/10	16/10		CirrusSpectralis	OAG	mRNFL, GCIPL, GCL, IPL		6 × 6
Aydogan et al. (39)	2017	Turkey	94	66	77	53.2 ± 11.1	59.4 ± 9.5	52.8 ± 8.2	57/37	36/30	62/15	RTVue	Mixed	pRNFL, GCC, TR, OR	3.45-mm circle	7 × 7
Akil et al. (38)	2017	USA	20	20		63.13 ± 16.43	65.375 ± 5.2		10/10	8/12		Cirrus	POAG	pRNFL	3.4-mm circle	
Kumar et al. (37)	2016	India	28	83		57.04 ± 2.78	61.2 ± 1.34		N/A	N/A		RTVue	Mixed	pRNFL, GCC	3.45-mm circle	6 × 6
Cennamo et al. (36)	2016	Italy	66	41		64.86 ± 7.12	63.78 ± 11.70		30/36	25/16		RTVue	OAG	pRNFL, GCC	3.45-mm circle	7 × 7
Park et al. (35)	2015	Korea	50	106		58.2 ± 13.7	56.0 ± 12.7		26/24	66/40		Cirrus	Mixed	pRNFL, GCIPL, TNM ratio	3.46-mm circle	6 × 6 × 2
Kim et al. (34)	2015	Korea	79	83		54.6 ± 11.8	57.5 ± 11.6		40/39	43/40		Cirrus	Mixed	pRNFL, GCIPL	Optic disc Cube 200 × 200	Macular Cube 512 × 128
Hwang et al. (33)	2015	Korea	48	110		51.7 ± 13.8	51.6 ± 12.7		N/A	N/A		Cirrus	Mixed	GCIPL		6 × 6
Yamada et al. (32)	2014	Japan	30	31		56.9 ± 14.7	61.8 ± 11.5		13/17	19/12		Spectralis	Mixed	mRNFL, GCC, GCL, TR		30 × 15 degreed macular area
Sung et al. (31)	2014	Korea	37	70		54.22 ± 12.70	53.97 ± 12.36		20/17	29/41		Cirrus	Mixed	pRNFL, GCIPL	3.46-mm circle	14.13 mm^2^ elliptical annulus area
Kim et al. (30)	2014	Korea	68	72		53.12 ± 10.69	56.83 ± 12.73		N/A	N/A		3D OCT-2000	OAG	pRNFL, mRNFL, GCIPL, GCC	3.46-mm circle	6 × 6
Kim et al. (29)	2014	Korea	103	111		57.2 ± 11.8.	56.8 ± 11.4		52/51	52/59		Cirrus	OAG	pRNFL, GCIPL	3.46-mm circle	Macular Cube 200 × 200
Holló et al. (48)	2014	Hungary	33		28	56.2 ± 12.1		50.7 ± 15.6	N/A		N/A	RTVue	Mixed	pRNFL, GCC, total retina thickness, G/T ratio	4-mm circle	
Arintawati et al. (28)	2013	Japan	32	81		58.94 ± 12.15	60.16 ± 16.77		18/14	45/36		RTVue	Mixed	pRNFL, GCC, FLV, GLV	3.5-mm circle	7 × 7
Pomorska et al. (47)	2012	Poland	33		27	61.0 ± 9.6		57.8 ± 11.2	17/16		18/9	Sratus	Mixed	pRNFL	3.4-mm circle	
Morooka et al. (27)	2012	Japan	23	24		56.8 ± 9.4	51.9 ± 12.2		16/7	13/11		RS3000	Mixed	pRNFL, GCC	3.45-mm circle	9 × 9
Horn et al. (26)	2011	Germany	77	52		59.2 ± 10.0	60.8 ± 10.5		41/36	27/25		Sepctralis	OAG	pRNFL	3.4-mm circle	
Garas et al. (46)	2011	Hungary	46		36	57.6 ± 11.8		51.5 ± 16.5	N/A		N/A	RTVue	Mixed	pRNFL, GCC, FLV	4-mm circle	
Taliantzis et al. (45)	2009	Greece	42		54	58.1 ± 11.6		56.7 ± 13.2	20/22		25/29	Stratus	Mixed	pRNFL	3.4-mm circle	

*PPG, pre-perimetric glaucoma; EG, early perimetric glaucoma; OHT, ocular hypertension; OAG, open-angle glaucoma; POAG, primary open-angle glaucoma; Mixed, unclassified glaucoma or more than one types of glaucoma; pRNFL, peripapillary retinal nerve fiber layer; mRNFL, macular retinal nerve fiber layer; GCIPL, ganglion cell plus inner plexiform layer; GCL, ganglion cell layer; IPL, inner plexiform layer; GCC, ganglion cell complex; G/T ratio, macular GCC to total retinal thickness; TR, macular total retinal parameters; OR, macular outer retinal parameters; FLV, focal loss volume; GLV, global loss volume; TNM ratio, temporal to nasal macular GCIPL thickness ratio; F/M, female/male; ONH, optic nerve head*.

**Table 2:** Methodological quality of included studies.

**Study**	**11-item check list recommended by AHRQ**												
	**i**	**ii**	**iii**	**iv**	**v**	**vi**	**vii**	**viii**	**ix**	**x**	**xi**	**Score**	**Quality**
Wang et al. (44)	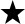	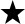	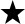			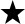		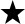		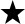		6	M
Sarigül Sezenöz et al. (43)	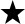	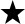	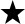			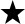		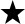				5	M
Lu et al. (42)	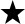	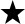	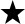			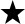		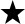		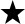		6	M
Hou et al. (41)	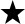	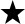	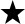			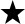		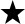		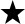		6	M
Kim et al. (40)	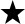	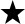	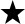			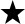		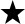		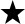		6	M
Aydogan et al. (39)	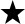	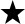	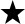			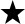		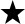		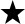		6	M
Akil et al. (38)	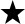	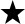	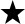			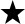	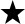	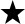		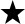		7	M
Kumar et al. (37)	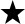	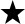	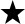			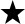	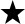	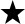		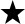		7	M
Cennamo et al. (36)	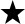	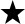	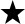				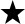	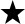		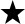		6	M
Park et al. (35)	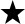	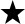	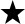		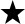	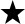	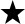	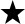		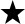		8	H
Kim et al. (34)	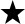	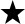	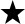			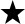	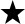	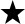		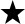		7	M
Hwang et al. (33)	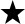	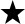				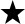		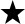		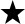		5	M
Yamada et al. (32)	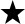	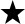	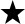		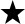	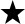	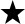	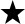		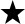		8	H
Sung et al. (31)	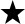	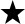	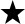			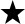	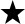	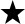		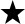		7	M
Kim et al. (30)	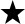	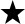	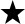		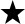	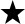	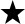	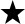		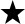		8	H
Kim et al. (29)	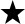	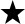	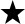			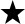		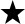		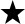		6	M
Holló et al. (48)	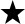	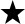	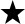			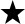		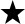		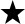		6	M
Arintawati et al. (28)	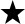	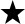	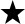					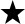		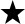		5	M
Pomorska et al. (47)	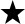	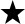	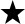			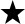	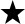			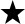		6	M
Morooka et al. (27)	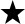	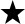	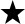		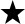	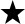		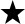		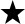		7	M
Horn et al. (26)	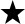	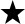	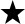			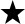		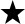		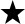		6	M
Garas et al. (46)	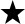	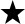	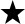			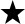	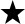	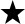		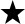		7	M
Taliantzis et al. (45)	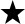	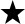	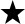			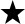				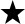		5	M

*AHRQ, Agency for Healthcare Research and Quality; H, high quality; M, moderate quality; L, low quality; high quality (score: 8–11); moderate quality (score: 4–7); low quality (score: 0–3). i, Define the source of information; ii, List inclusion and exclusion criteria for exposed and unexposed subjects (cases and controls) or refer to previous publications; iii, Indicate time period used for identifying patients; iv, Indicate whether or not subjects were consecutive if not population-based; v, Indicate if evaluators of subjective components of study were masked to other aspects of the status of the participants; vi, Describe any assessments undertaken for quality assurance purposes; vii, Explain any patient exclusions from analysis; viii, Describe how confounding was assessed and/or controlled; ix, If applicable, explain how missing data were handled in the analysis; x, Summarize patient response rates and completeness of data collection; xi, Clarify what follow-up, if any, was expected and the percentage of patients for which incomplete data or follow-up was obtained. 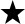
means the study meets the requirements of the corresponding items*.

### PPG vs. EG

#### Peripapillary RNFL Thickness

**Figure 2:**
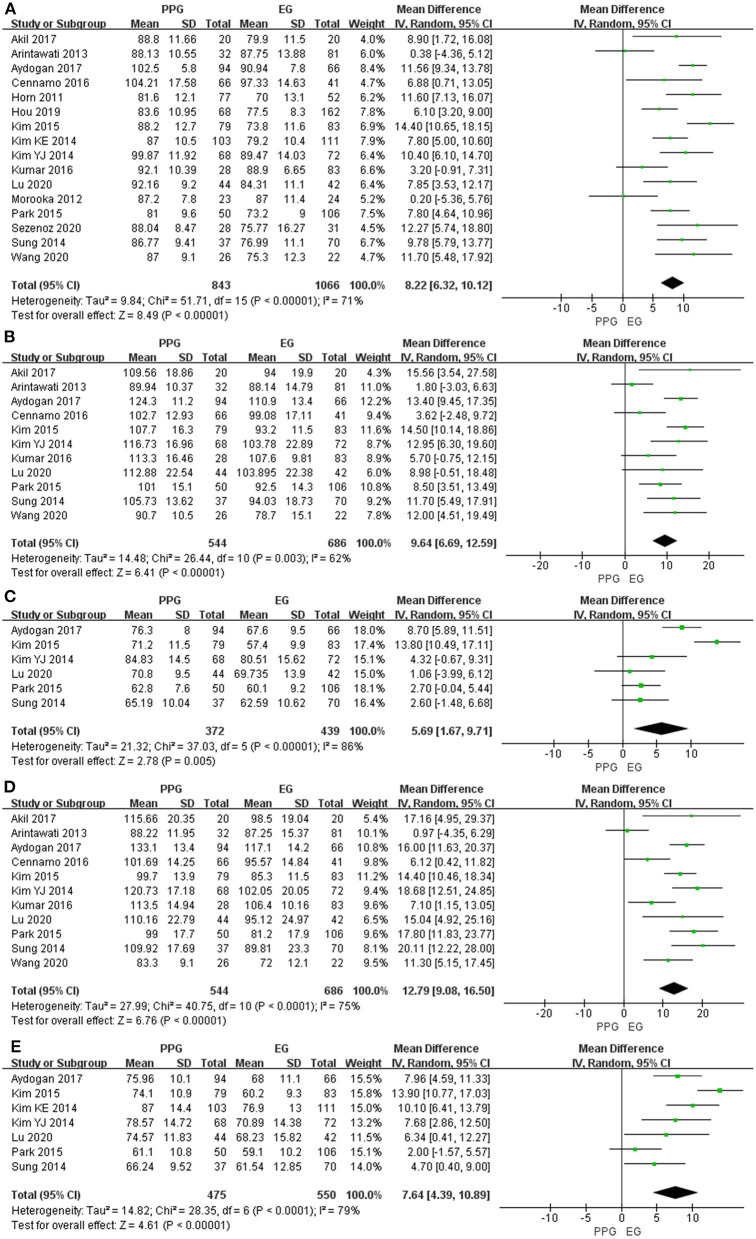
pRNFL thickness in patients with PPG and EG. **(A)** Average. **(B)** Superior. **(C)** Nasal. **(D)** Inferior. **(E)** Temporal.

Sixteen studies evaluating pRNFL thickness showed significant heterogeneity (I^2^ > 50%); thus, the random-effects model was used. The pooled results demonstrated a significant decrease in the average and quadrant pRNFL thickness in the EG eyes compared with the PPG eyes (average: WMD = 8.22, 95% CI = 6.32–10.12, *P* < 0.00001, Figure 2A; superior: WMD = 9.64, 95% CI = 6.69–12.59, *P* < 0.00001, Figure 2B; nasal: WMD = 5.69, 95% CI = 1.67–9.71, *P* = 0.005, Figure 2C; inferior: WMD = 12.79, 95% CI = 9.08–16.50, *P* < 0.00001, Figure 2D; temporal: WMD = 7.64, 95% CI = 4.39–10.89, *P* < 0.00001, Figure 2E).

#### Macular GCIPL Thickness

**Figure 3:**
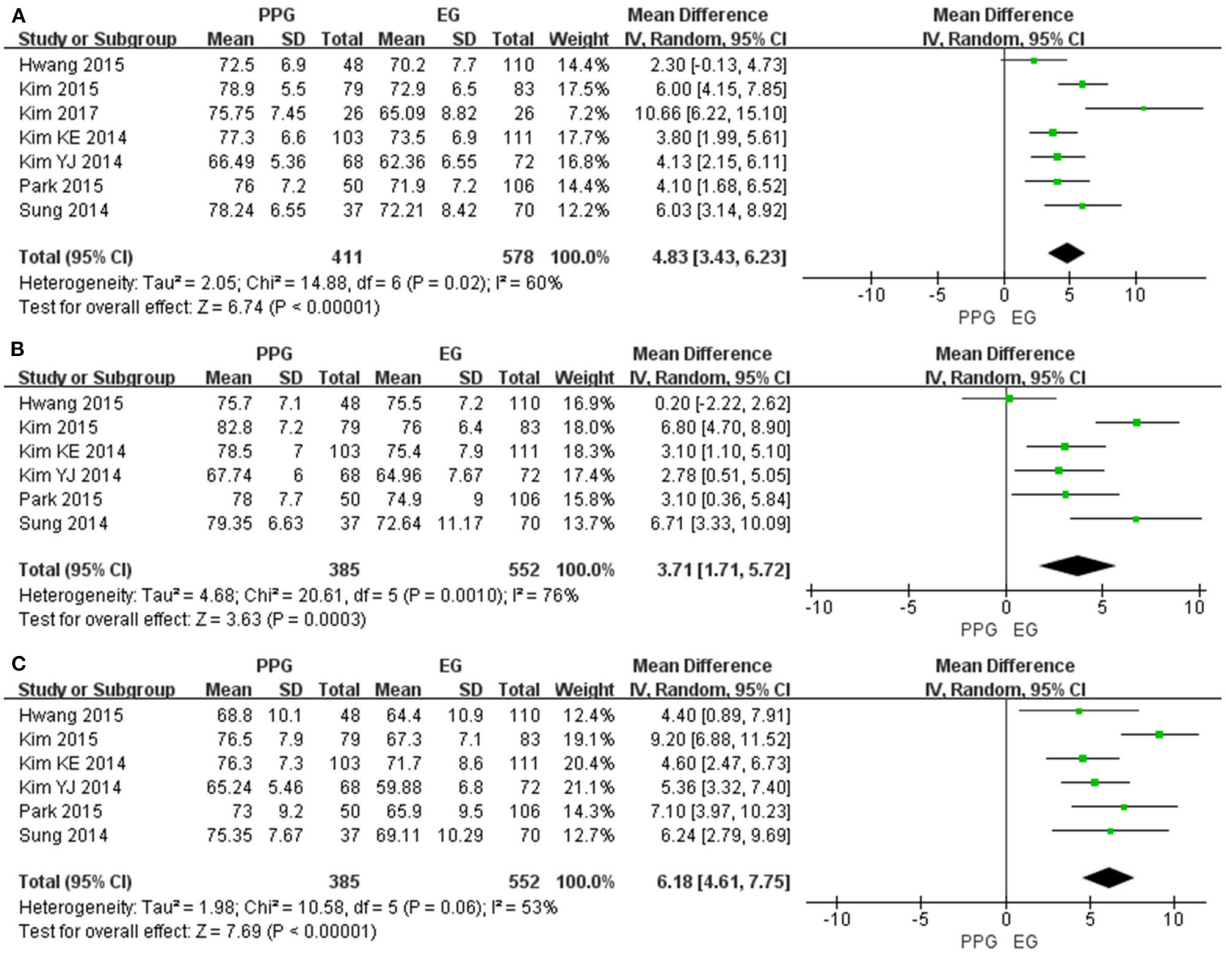
mGCIPL thickness in patients with PPG and EG. **(A)** Average. **(B)** Superior. **(C)** Inferior.

Seven of the included studies assessed the average mGCIPL thickness and six studies assessed superior and inferior mGCIPL thickness with significant heterogeneity (I^2^ > 50%). The pooled results indicated the average mGCIPL thickness was significantly less in the EG eyes than in the PPG eyes (WMD = 4.83, 95% CI = 3.43–6.23, *P* < 0.00001, Figure 3A). Likewise, the superior and inferior mGCIPL were also significantly thinner in patients with EG than in those with PPG (superior: WMD = 3.71, 95% CI = 1.71–5.72, *P* = 0.0003, Figure 3B; inferior: WMD = 6.18, 95% CI = 4.61–7.75, *P* < 0.00001, Figure 3C).

#### Macular GCC Thickness

**Figure 4:**
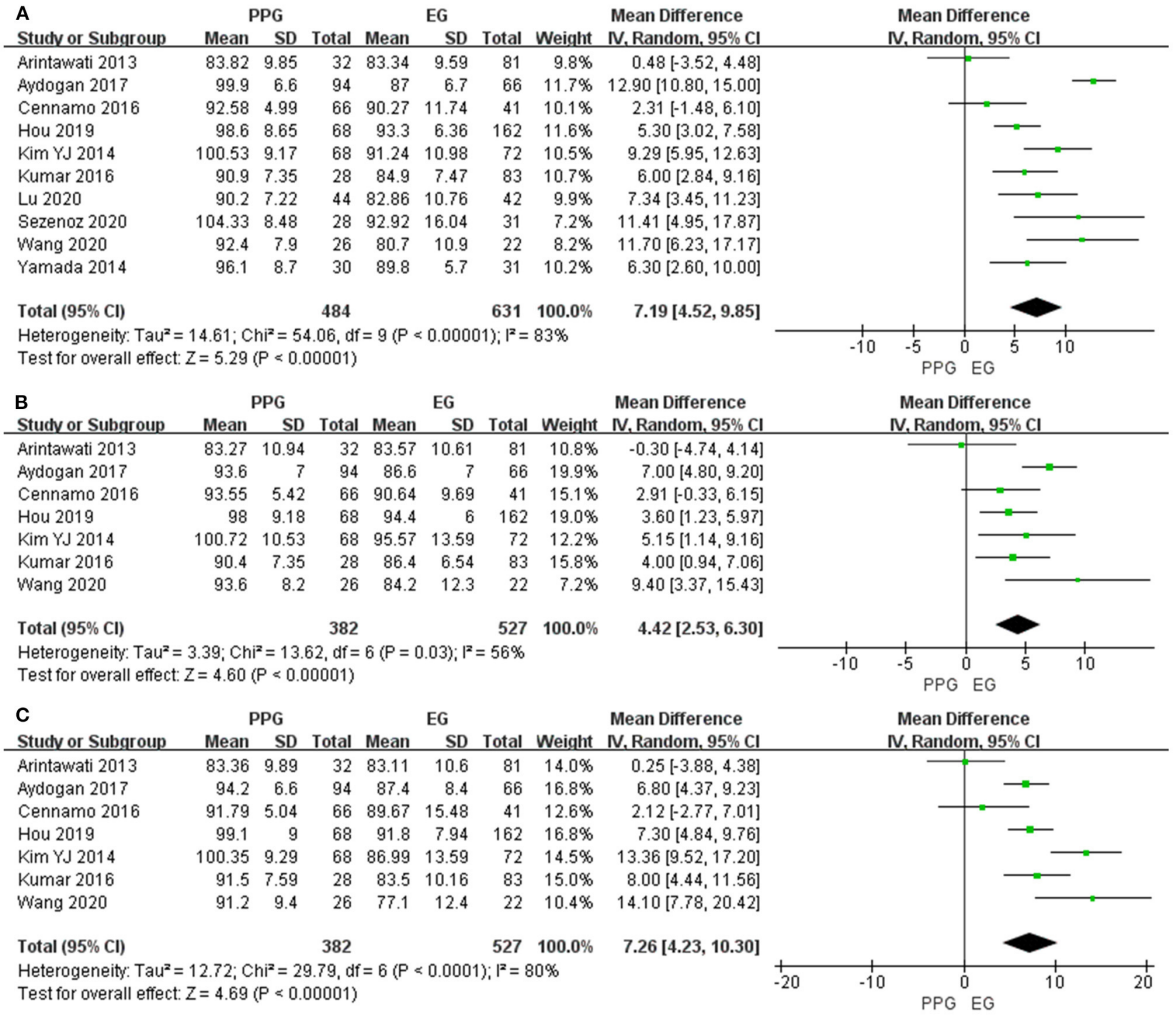
mGCC thickness in patients with PPG and EG. **(A)** Average. **(B)** Superior. **(C)** Inferior.

Ten studies measured the average mGCC thickness and seven studies assessed quadrant mGCC thickness with I^2^ > 50%. All the pooled mGCC thickness values were significantly reduced in the EG eyes compared to the PPG eyes (average: WMD = 7.19, 95% CI = 4.52–9.85, *P* < 0.00001, Figure 4A; superior: WMD = 4.42, 95% CI = 2.53–6.30, *P* < 0.00001, Figure 4B; inferior: WMD = 7.26, 95% CI = 4.23–10.30, *P* < 0.00001, Figure 4C).

#### Subgroup Analyses

The stratified subgroup analysis according to the type of glaucoma (Table 3) showed a similar decreased trend of pRNFL and mGCIPL thickness in EG compared with PPG except for the superior quadrant of pRNFL in the open-angle glaucoma subgroup (WMD = 8.19, 95% CI = −0.95 to 17.33, *P* = 0.08). However, unlike the combined pooled data, in the subgroup of the open-angle glaucoma in terms of the average and inferior mGCC thickness, there was no difference between the EG and PPG eyes (average: WMD = 5.86, 95% CI = −0.98 to 12.70, *P* = 0.09; inferior: WMD = 7.85, 95% CI = −3.17 to 18.86, *P* = 0.16).

**Table 3:** Subgroup analysis of average and sectoral pRNFL, mGCIPL and mGCC thickness according to the type of glaucoma in patients with PPG and EG.

**Subgroup**	**No**.	**Heterogeneity**		**WMD (95% CI)**	**Overall effect**	
		**I^**2**^**	***P***		**Z**	***P***
**1. pRNFL**						
**POAG**						
Average	4	0%	0.42	7.45 (5.30, 9.59)	6.81	<0.00001
Superior	3	0%	0.70	11.75 (6.47, 17.04)	4.36	<0.0001
Inferior	3	0%	0.64	13.07 (8.24, 17.89)	5.31	<0.00001
**OAG**						
Average	4	0%	0.41	8.99 (7.02, 10.96)	8.94	<0.00001
Superior	2	76%	0.04	8.19 (−0.95, 17.33)	1.76	**0.08**
Inferior	2	88%	0.003	12.34 (0.03, 24.65)	1.97	0.05
**Mixed**						
Average	8	84%	<0.00001	7.60 (4.13, 11.07)	4.29	<0.0001
Superior	6	75%	0.001	9.41 (5.29, 13.53)	4.48	<0.00001
Inferior	6	83%	<0.0001	12.47 (6.41, 18.52)	4.03	<0.0001
**2. mGCIPL**						
**OAG**						
Average	3	75%	0.02	5.44 (2.57, 8.31)	3.72	0.0002
Superior	2	0%	0.84	2.96 (1.46, 4.46)	3.87	0.0001
Inferior	2	0%	0.61	5.00 (3.52, 6.47)	6.65	<0.00001
**Mixed**						
Average	4	55%	0.08	4.63 (2.87, 6.40)	5.14	<0.00001
Superior	4	84%	0.0003	4.16 (0.87, 7.45)	2.48	0.01
Inferior	4	46%	0.13	7.32 (5.84, 8.80)	9.66	<0.00001
**3. mGCC**						
**POAG**						
Average	3	58%	0.09	7.38 (4.09, 10.67)	4.40	<0.0001
Superior	2	68%	0.08	5.81 (0.29, 11.33)	2.06	0.04
Inferior	2	74%	0.05	10.05 (3.51, 16.59)	3.01	0.003
**OAG**						
Average	2	86%	0.007	5.86 (−0.98, 12.70)	1.68	**0.09**
Superior	2	0%	0.39	3.79 (1.27, 6.32)	2.95	0.003
Inferior	2	92%	0.0004	7.85 (−3.17, 18.86)	1.40	**0.16**
**Mixed**						
Average	5	89%	<0.00001	7.37 (2.64, 12.11)	3.05	0.002
Superior	3	78%	0.01	3.96 (0.12, 7.80)	2.02	0.04
Inferior	3	78%	0.01	5.23 (1.14, 9.32)	2.51	0.01

*PPG, pre-perimetric glaucoma; EG, early perimetric glaucoma; POAG, primary open-angle glaucoma; OAG, open-angle glaucoma; Mixed, unclassified glaucoma or more than one types of glaucoma; pRNFL, peripapillary retinal nerve fiber layer; mGCIPL, macular ganglion cell plus inner plexiform layer; mGCC, macular ganglion cell complex; WMD, weighted mean difference; CI, confidence interval; I^2^, I-square heterogeneity statistic; Z, Z-statistic. The bold values refer to the studies with a P value > 0.05*.

In addition, considering the different types of OCT (Table 4), the average and quadrant pRNFL thickness were significantly lower in the EG eyes than in the PPG eyes regardless of the kind of OCT that was used except for the nasal quadrant of pRNFL in the Cirrus SD-OCT subgroup (WMD = 6.38, 95% CI = −1.02 to 13.78, *P* = 0.09).

**Table 4:** Subgroup analysis of average and sectoral pRNFL thickness according to the type of OCT in patients with PPG and EG.

**Subgroup**	**No**.	**Heterogeneity**		**WMD (95% CI)**	**Overall effect**	
		**I^**2**^**	***P***		**Z**	***P***
**RTVue SD-OCT**						
Average	6	81%	<0.0001	6.96 (2.96, 10.95)	3.42	0.0006
Superior	6	71%	0.004	7.51 (2.98, 12.04)	3.25	0.001
Nasal	2	0%	1.00	8.70 (6.72, 10.68)	8.60	<0.00001
Inferior	6	77%	0.0006	9.18 (4.13, 14.22)	3.56	0.0004
Temporal	2	0%	0.64	7.56 (4.64, 10.49)	5.06	<0.00001
**Cirrus SD-OCT**						
Average	5	56%	0.06	9.67 (7.09, 12.25)	7.35	<0.00001
Superior	4	14%	0.32	12.04 (8.91, 15.17)	7.53	<0.00001
Nasal	3	93%	0.00001	6.38 (-1.02, 13.78)	1.69	**0.09**
Inferior	4	0%	0.56	16.18 (13.24, 19.13)	10.77	<0.00001
Temporal	4	89%	<0.00001	7.74 (2.20, 13.29)	2.74	0.006

*PPG, pre-perimetric glaucoma; EG, early perimetric glaucoma; pRNFL, peripapillary retinal nerve fiber layer; OCT, optical coherence tomography; SD-OCT, spectral domain OCT; WMD, weighted mean difference; CI, confidence interval; I^2^, I-square heterogeneity statistic; Z, Z-statistic. The bold value refers to the study with a P value > 0.05*.

The subgroup analysis regarding the scan area of the macular region (Table 5) revealed that the average and sectoral mGCC thickness were significantly lower in patients with EG than in those with PPG in the 6 × 6 mm subgroup, whereas no difference was found in 7 × 7 mm subgroup (average: WMD = 5.36, 95% CI = −3.27 to 13.99, *P* = 0.22; superior: WMD = 3.54, 95% CI = −0.64 to 7.72, *P* = 0.10; inferior: WMD = 3.35, 95% CI = −1.08 to 7.79, *P* = 0.14).

**Table 5:** Subgroup analysis of average and sectoral mGCC thickness according to the macular scan area (mm^2^) in patients with PPG and EG.

**Subgroup**	**No**.	**Heterogeneity**		**WMD (95% CI)**	**Overall effect**	
		**I^**2**^**	***P***		**Z**	***P***
**6** **×** **6**						
Average	4	25%	0.26	8.09 (5.91, 10.27)	7.27	<0.00001
Superior	3	18%	0.29	5.28 (2.69, 7.86)	4.00	<0.0001
Inferior	3	61%	0.08	11.44 (7.38, 15.50)	5.53	<0.00001
**7** **×** **7**						
Average	3	95%	<0.00001	5.36 (−3.27, 13.99)	1.22	**0.22**
Superior	3	80%	0.006	3.54 (−0.64, 7.72)	1.66	**0.10**
Inferior	3	76%	0.01	3.35 (−1.08, 7.79)	1.48	**0.14**

*PPG, pre-perimetric glaucoma; EG, early perimetric glaucoma; mGCC, macular ganglion cell complex; WMD, weighted mean difference; CI, confidence interval; I^2^, I-square heterogeneity statistic; Z, Z-statistic. The bold values refer to the studies with a P value > 0.05*.

### PPG vs. OHT

#### Peripapillary RNFL Thickness

**Figure 5:**
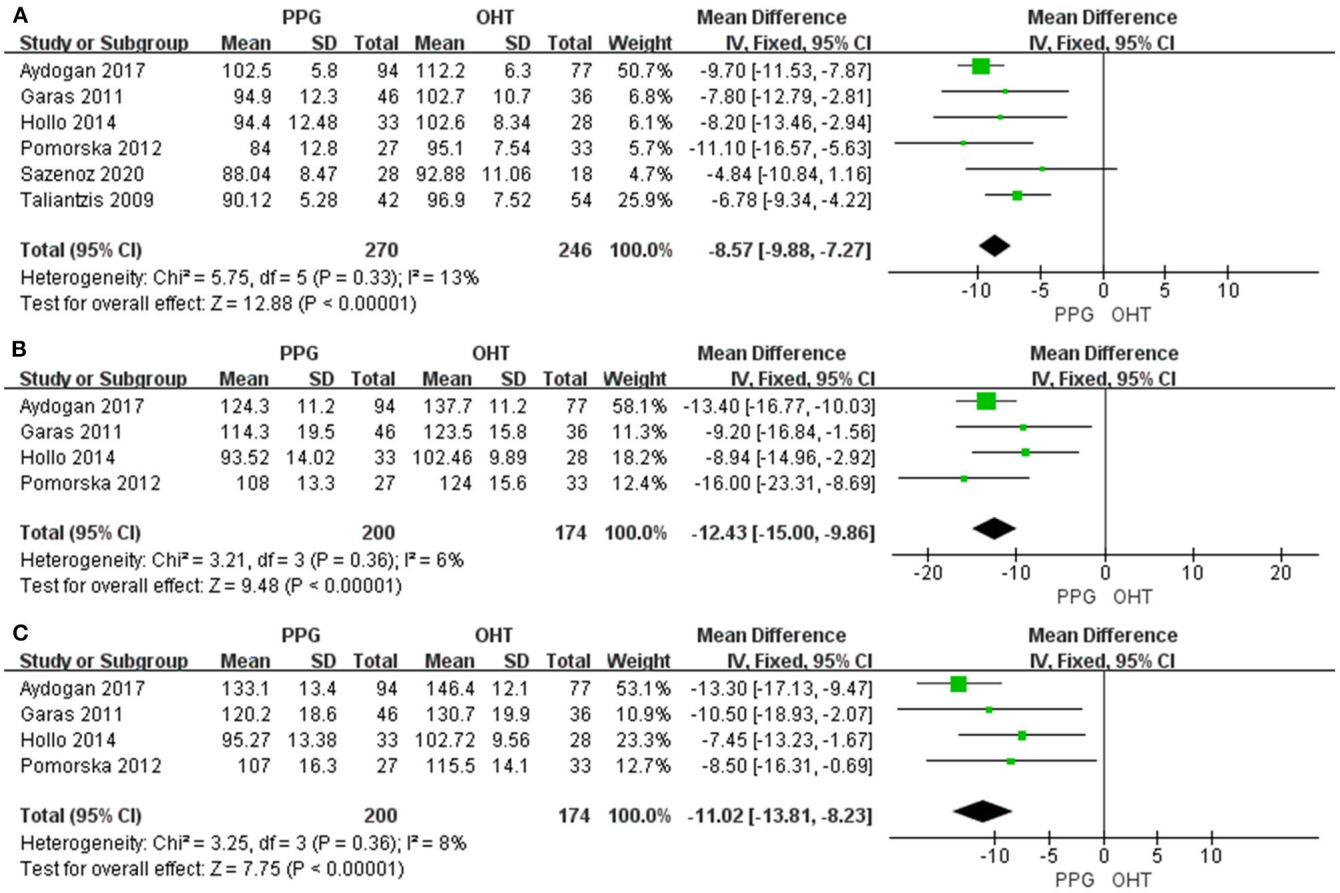
pRNFL thickness in patients with PPG and OHT. **(A)** Average. **(B)** Superior. **(C)** Inferior.

Six studies evaluated average pRNFL thickness in patients with PPG and OHT with I^2^ < 50%; thus, fixed-effects model was used. The pooled results demonstrated that the average and quadrant pRNFL thickness were significantly lower in patients with PPG than in those with OHT (average: WMD = −8.57, 95% CI = −9.88 to −7.27, *P* < 0.00001, Figure 5A; superior: WMD = −12.43, 95% CI = −15.00 to −9.86, *P* < 0.00001, Figure 5B; inferior: WMD = −11.02, 95% CI = −13.81 to −8.23, *P* < 0.00001, Figure 5C).

#### Macular GCC Thickness

**Figure 6:**
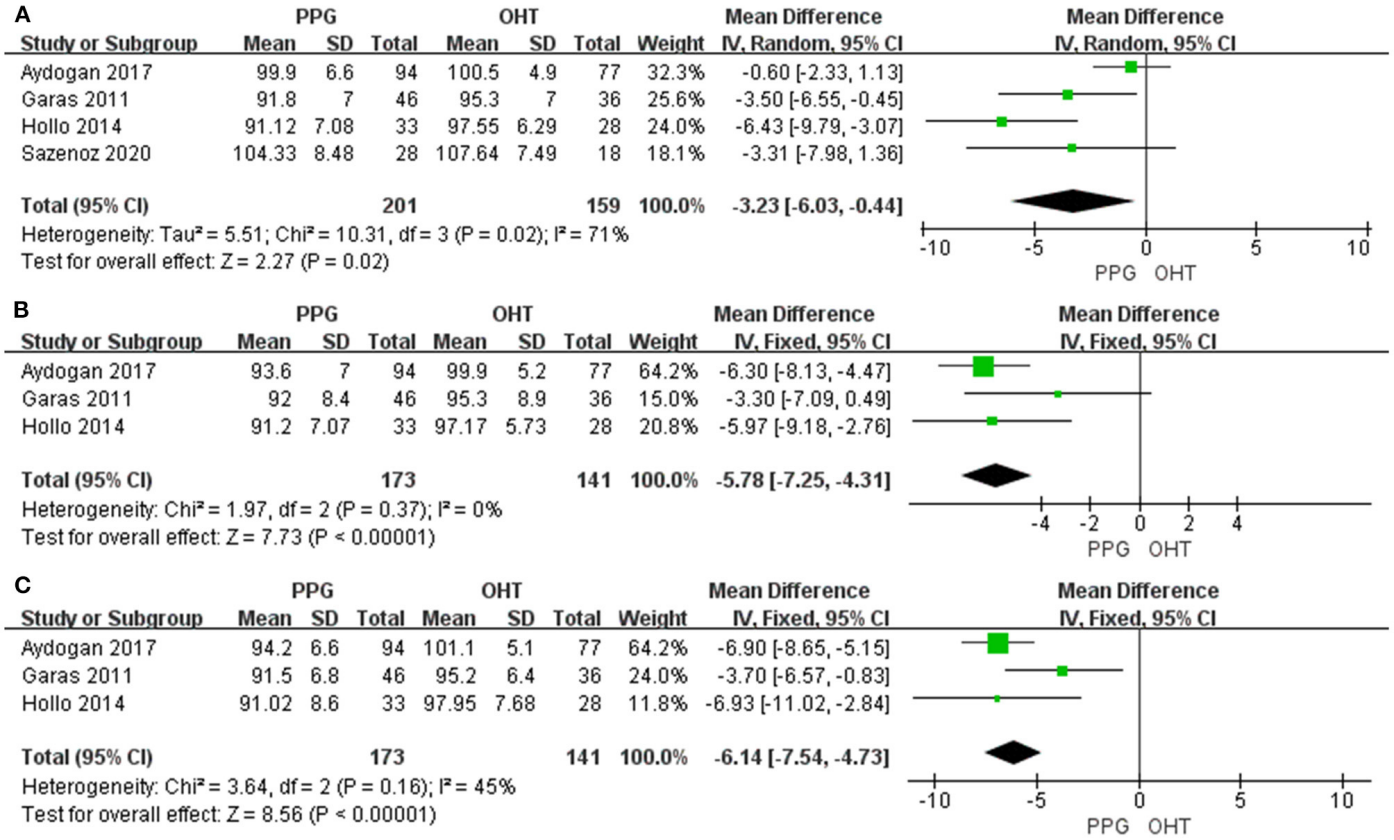
mGCC thickness in patients with PPG and OHT. **(A)** Average. **(B)** Superior. **(C)** Inferior.

Four studies measured average mGCC thickness with I^2^ > 50%, and three studies assessed sectoral mGCC thickness with I^2^ < 50%. The average and sectoral mGCC were significantly thinner in the PPG eyes than those in the OHT eyes (average: WMD = −3.23, 95% CI = −6.03 to −0.44, *P* = 0.02, Figure 6A; superior: WMD = −5.78, 95% CI = −7.25 to −4.31, *P* < 0.00001, Figure 6B; inferior: WMD = −6.14, 95% CI = −7.54 to −4.73, *P* < 0.00001, Figure 6C).

#### Subgroup Analysis

The subgroup analysis demonstrated that the average pRNFL thickness was significantly lower in patients with PPG than in those with OHT, regardless of the type of OCT (SD-OCT: WMD = −9.04, 95% CI = −10.62 to −7.46, *P* < 0.00001; TD-OCT: WMD = −7.56, 95% CI = −9.88 to −5.24, *P* < 0.00001, Supplementary Figure 1).

#### Publication Bias

No significant publication bias was shown according to the results of Egger's and Begg's tests (*P* > 0.05, Table 6), and no obvious asymmetry or correlation between study and effect size was observed in the funnel plot in terms of pRNFL and mGCC thickness (Supplementary Figures 2–5). However, slight asymmetry was noted in the funnel plot of average mGCIPL thickness (Supplementary Figure 6A), but not in those of superior and inferior mGCIPL thickness (Supplementary Figures 6B,C).

**Table 6:** Begg's and Egger's tests results for the evaluation of publication bias.

**Outcome indicators**	**No**.	**Begg's test**		**Egger's test**	
		**z**	**Pr > |z|**	***t***	***P* > |*t*|**
**1. PPG vs. EG**					
**pRNFL**					
Average	16	0.23	0.822	−0.55	0.589
Superior	11	0.62	0.533	−0.53	0.606
Nasal	6	0.00	1.000	−1.21	0.292
Inferior	11	1.71	0.087	−0.76	0.465
Temporal	7	0.30	0.764	−0.60	0.573
**mGCC**					
Average	10	1.07	0.283	0.20	0.849
Superior	7	0.00	1.000	0.04	0.973
Inferior	7	0.00	1.000	−0.11	0.920
**mGCIPL**					
Average	7	1.20	0.230	1.50	0.195
Superior	6	0.38	0.707	0.26	0.806
Inferior	6	0.00	1.000	0.36	0.735
**2. PPG vs. OHT**					
**pRNFL**					
Average	6	1.13	0.260	2.71	0.053
Superior	4	−0.34	1.000	0.85	0.487
Inferior	4	0.34	0.734	3.32	0.08
**mGCC**					
Average	4	0.34	0.734	−1.84	0.206
Superior	3	0.00	1.000	0.51	0.700
Inferior	3	0.00	1.000	1.10	0.471

*PPG, pre-perimetric glaucoma; EG, early perimetric glaucoma; OHT, ocular hypertension; pRNFL, peripapillary retinal nerve fiber layer; mGCC, macular ganglion cell complex; mGCIPL, macular ganglion cell plus inner plexiform layer*.

#### Sensitivity Analyses

There was no obvious change in the results after “leave-one-out” sensitivity analyses, indicating the reproducibility and stability of our results (Supplementary Figures 7–11). However, the sensitivity analysis of average mGCIPL thickness in patients with PPG and EG indicated that the study by Kim et al. (40) contributed mostly to the heterogeneity (Table 7). After excluding this study, heterogeneity was largely reduced (I^2^ = 34%, Supplementary Figure 12) and the funnel plot became symmetrical (Table 6 and Supplementary Figure 13, *P*-value of Egger's test increased from 0.195 to 0.745). In addition, the sensitivity analysis of average mGCC thickness in PPG eyes and EG eyes demonstrated that the study by Aydogan et al. (39) was the major source of the heterogeneity (Table 8). After excluding this study, the I^2^ decreased from 83% to 64% (Supplementary Figure 14). In the sensitivity analysis of average mGCC thickness in the PPG eyes and OHT eyes, the study by Aydogan et al. (39) was also shown to introduce the heterogeneity mostly (Table 9). After excluding this study, no heterogeneity was noted (I^2^ = 0%, Supplementary Figure 15).

**Table 7:** Sensitivity analysis of average mGCIPL thickness in patients with PPG and EG.

**Study excluded**	**Fixed-effects model**		**Random-effects model**		**Heterogeneity**
	**WMD (95% CI)**	***P***	**WMD (95% CI)**	***P***	**I^**2**^**
**Kim et al**. **(****40****)**	**4.40 (3.53, 5.27)**	**<0.00001**	**4.39 (3.30, 5.47)**	**<0.00001**	**34%**
Park et al. (35)	4.71 (3.80, 5.63)	<0.00001	5.00 (3.35, 6.65)	<0.00001	66%
Kim et al. (34)	4.26 (3.30, 5.23)	<0.00001	4.60 (3.02, 6.18)	<0.00001	59%
Hwang et al. (33)	4.97 (4.05, 5.88)	<0.00001	5.21 (3.80, 6.62)	<0.00001	54%
Sung et al. (31)	4.50 (3.60, 5.40)	<0.00001	4.68 (3.12, 6.24)	<0.00001	64%
Kim et al. (30)	4.75 (3.80, 5.70)	<0.00001	5.03 (3.32, 6.73)	<0.00001	66%
Kim et al. (29)	4.87 (3.90, 5.85)	<0.00001	5.10 (3.41, 6.78)	<0.00001	64%

*PPG, pre-perimetric glaucoma; EG, early perimetric glaucoma; mGCIPL, macular ganglion cell plus inner plexiform layer; WMD, weighted mean difference; CI, confidence interval; I^2^, I-square heterogeneity statistic. The bold values refer to the study that has contributed mostly to the heterogeneity*.

**Table 8:** Sensitivity analysis of average mGCC thickness in patients with PPG and EG.

**Study excluded**	**Fixed-effects model**		**Random-effects model**		**Heterogeneity**
	**WMD (95% CI)**	***P***	**WMD (95% CI)**	***P***	**I^**2**^**
Wang et al. (44)	7.51 (6.46, 8.56)	<0.00001	6.78 (3.98, 9.58)	<0.00001	85%
Sarigül Sezenöz et al. (43)	7.56 (6.52, 8.61)	<0.00001	6.85 (4.06, 9.65)	<0.00001	85%
Lu et al. (42)	7.69 (6.61, 8.76)	<0.00001	7.17 (4.23, 10.11)	<0.00001	85%
Hou et al. (41)	8.27 (7.11, 9.43)	<0.00001	7.44 (4.43, 10.45)	<0.00001	84%
**Aydogan et al**. **(****39****)**	**5.98 (4.80, 7.17)**	**<0.00001**	**6.28 (4.20, 8.37)**	**<0.00001**	**64%**
Kumar et al. (37)	7.86 (6.77, 8.95)	<0.00001	7.33 (4.36, 10.31)	<0.00001	85%
Cennamo et al. (36)	8.09 (7.02, 9.16)	<0.00001	7.73 (5.00, 10.45)	<0.00001	83%
Yamada et al. (32)	7.78 (6.70, 8.85)	<0.00001	7.29 (4.35, 10.23)	<0.00001	85%
Kim et al. (30)	7.49 (6.40, 8.57)	<0.00001	6.95 (3.98, 9.91)	<0.00001	85%
Arintawati et al. (28)	8.17 (7.10, 9.24)	<0.00001	7.90 (5.34, 10.46)	<0.00001	80%

*PPG, pre-perimetric glaucoma; EG, early perimetric glaucoma; mGCC, macular ganglion cell complex; WMD, weighted mean difference; CI, confidence interval; I^2^, I-square heterogeneity statistic. The bold values refer to the study that has contributed mostly to the heterogeneity*.

**Table 9:** Sensitivity analysis of average mGCC thickness in patients with PPG and OHT.

**Study excluded**	**Fixed-effects model**		**Random-effects model**		**Heterogeneity**
	**WMD (95% CI)**	***P***	**WMD (95% CI)**	***P***	**I^**2**^**
Sarigül Sezenöz et al. (43)	−2.16 (−3.53,−0.79)	0.002	−3.28 (−6.75, 0.19)	0.06	80%
**Aydogan et al**. **(****39****)**	**−4.54 (−6.57**, **−2.51)**	**<0.0001**	**−4.54 (−6.57**, **−2.51)**	**<0.0001**	**0%**
Holo et al. (48)	−1.49 (−2.92, −0.06)	0.04	−1.93 (−4.05, 0.19)	0.08	39%
Garas et al. (46)	−1.96 (−3.42, −0.51)	0.008	−3.52 (−7.20, 0.69)	0.11	79%

*PPG, pre-perimetric glaucoma; OHT, ocular hypertension; mGCC, macular ganglion cell complex; WMD, weighted mean difference; CI, confidence interval; I^2^, I-square heterogeneity statistic. The bold values refer to the study that has contributed mostly to the heterogeneity*.

## Discussion

In the present study, we first pooled the average and sectoral pRNFL, mGCIPL, and mGCC thickness in patients with PPG and EG. Our results demonstrated that the average and sectoral pRNFL, mGCIPL, and mGCC were significantly thinner in the EG eyes than in the PPG eyes. These findings were consistent across several studies (26, 29, 34, 38–41, 43, 44), whereas the results of eight studies were not significantly different in terms of the average or sectoral pRNFL thickness (27, 28, 30, 31, 35–37, 42); two studies reported there was no significant difference in the EG eyes compared to the PPG eyes concerning mGCC thickness (28, 36), and one study did not demonstrate a significant reduction in the EG eyes regarding the average and superior mGCIPL thickness (33). Currently, the exact biomechanisms of glaucomatous neurodegeneration remain poorly understood (1, 55). Nevertheless, continuous and progressive glaucomatous damage may lead to configuration changes in retinal ganglion cell dendrites, soma, and axons (56, 57), causing the attenuation of thickness in corresponding residing sites, inner plexiform layer, ganglion cell layer and RNFL (20). Our pooled results of pRNFL, mGCIPL, and mGCC thickness suggested that more serious structural damage occurred in EG than in PPG.

To explore the source of heterogeneity across the included studies, we performed subgroup analyses according to the different types of glaucoma. Our findings did not show a significant decrease in the superior quadrant of pRNFL (*P* = 0.08), and the average, as well as the inferior mGCC thickness in the EG eyes when patients with open-angle glaucoma were enrolled (average: *P* = 0.09; inferior quadrant: *P* = 0.16). This was probably mainly due to the relatively small sample size in the subgroup of OAG regarding the pRNFL and the mGCC thickness (*N* = 2), because of which heterogeneity could not be excluded. Another reason was that no quality assurance step was taken in the study of Cennamo et al. (36). Since only a single OCT examination was performed to evaluate each parameter by one experienced ophthalmologist, ensuring the reproducibility and reliability of the examination results was difficult.

Based on the anatomy of retina, ~30–50% RGCs are centered within the 4.5-mm-circle region of the fovea (44, 58–60); thus, using a relatively small scan area of 3 × 3 mm may not allow the differentiation of EG from PPG or suspected glaucoma (41, 61). For this reason, we also performed a subgroup analysis of the macular scan area. Interestingly, our pooled data showed that when the 6 × 6 mm scan protocol was used, there was a significant decrease in mGCC thickness in the EG eyes compared with the PPG eyes, whereas there was no difference when the 7 × 7 mm scan protocol was used. One reason for the different diagnostic performances between these two scan protocols may be the decreased signal-to-noise ratio. Although the enlarged scan area could cover the region with the most abundant RGCs, the concomitant decrease in signal-to-noise ratio and increase in test-retest variability (62) may have undervalued the assessment of GCC thickness measured by SD-OCT. Another explanation was the strict inclusion criteria by Arintawati et al. (28). In this study, investigators only accepted subjects with all the typical glaucomatous changes observed in fundus photographs to avoid false-positive cases. Consequently, patients with higher severity may have been enrolled; thus, showing a minimal difference between the PPG and EG groups. Unfortunately, because of the small sample size (*N* = 3), we were unable to include other scan areas and protocols, introducing difficulties in the overall evaluation of the diagnostic values regarding different macular scan protocols. In addition, sensitivity analysis demonstrated that the study of Aydogan et al. (39) contributed mostly to the heterogeneity of average mGCC thickness. The main reason was that the age was not well-matched among PPG and EG groups (*P* < 0.001), which may induce potential bias since glaucoma is an age-related optic neuropathy (55). However, after excluding this study, the heterogeneity decreased (I^2^ decreased from 83 to 64%).

Recently, in addition to OCT, several studies have focused on the macular microvasculature changes in PPG and EG via OCT angiography, revealing the progression patterns of glaucoma with respect to microvascular dysregulation (41, 42, 44, 63, 64). Although macular vessel density (VD) was reported to significantly decrease both in PPG and EG, one study demonstrated that mGCC thickness, unlike macular VD, could serve as a tool to discriminate PPG from EG (41). The study also showed that the percentage loss of mGCC thickness was significantly higher than that of macular VD both in PG and EG. Another study reported that both inferotemporal and superotemporal pRNFL thickness were significantly decreased in EG eyes compared to PPG eyes whereas only the inferotemporal sector of the radial peripapillary capillary VD experienced a significant decrease (42). Considering previous OCT studies that suggest that structural deterioration usually occurs before functional loss (10–18), these findings consolidate the results of our study, which indicate that the OCT evaluation of macular structure changes could help to clarify the pathophysiological mechanisms of glaucoma and differentiate PPG from EG.

In a subgroup analysis of pRNFL thickness according to the type of SD-OCT, the pooled results were generally consistent with the combined pooled data except for the nasal quadrant in the Cirrus SD-OCT subgroup. The main reason was that Kim et al. (34) utilized the optic disc Cube 200 × 200 scanning protocol, whereas the other two studies focused on a 3.46-mm-diameter circle region of the ONH (31, 35). Although all types of SD-OCT could detect characteristic glaucomatous damage patterns of pRNFL thickness, different algorithms, software, and parameters may have induced subtle differences in diagnostic performance (65). However, our pooled results demonstrate the important diagnostic value of SD-OCT in evaluating the severity of the glaucomatous damage of the ONH.

Apart from this, studies have shown that individuals with OHT are at higher risk of developing glaucoma than others (3, 4, 66); however, there is no optimal IOP cut-off that possesses both reasonable sensitivity and specificity (3), which may delay the early diagnosis until patients are found to have apparent optic disc structural deterioration. Although the widely used cut-off of 21 mmHg has high sensitivity, it can decrease the specificity to 44% (67). In addition, spectrum bias usually occurs when studies inappropriately include the control groups without any suspicious symptoms of the disease, which can impair the diagnostic efficacy when clinically non-relevant individuals are enrolled (68–70). Thus, we also pooled the average and sectoral pRNFL and mGCC thickness in patients with PPG and OHT.

The pooled results revealed that the pRNFL and mGCC were significantly thinner in the PPG eyes than those in the OHT eyes, which were consistent with several investigations (45, 47, 48). However, two studies reported that there was no difference in patients with PPG and OHT in terms of the average mGCC thickness (39, 43); one study demonstrated that the average pRNFL thickness could not serve as a valued indicator for differential diagnosis (43), and another study showed that no difference was noted in the PPG eyes and OHT eyes regarding the superior mGCC thickness (46).

Similar to the investigation of exploring the heterogeneity across different OCT parameters for the differential diagnosis of PPG and EG, we also performed subgroup analysis of average pRNFL thickness according to the type of OCT. However, no difference was noted in the pooled results with either SD-OCT or TD-OCT. Compared to the traditional TD-OCT, SD-OCT is the latest generation of OCT with ultra-high scanning speed and retinal image resolutions, and is reported to have higher diagnostic abilities in terms of sectoral pRNFL and macular thickness. However, both SD-OCT and TD-OCT showed comparable reproducibility regarding the mean pRNFL (71). This was consistent with our finding. In the sensitivity analysis, the study of Aydogan et al. (39) also contributed mostly to the heterogeneity of mGCC thickness in patients with PPG and OHT. This may be due to usage of the different macular scanning protocol.

Despite the strengths of our study, some of its limitations should be considered. First, mild asymmetry was shown in the funnel plot of average mGCIPL thickness (Supplementary Figure 6A), suggesting potential publication bias. To elucidate the source of the bias, we performed a “leave-one-out” sensitivity analysis (Table 7). The results showed that the study by Kim et al. (40) contributed mostly to the heterogeneity in average mGCIPL thickness, wherein some problems of automated segmentation software occurred, although measures were taken to minimize the consequence of the segmentation error. Therefore, after excluding this study, low heterogeneity (I^2^ = 34%) was noted (Supplementary Figure 12), and the funnel plot became symmetrical (Supplementary Figure 13). Second, regarding the subgroup analysis of the macular scan area, due to the relatively small sample size, we did not include other scan protocols apart from the 6 × 6 mm and 7 × 7 mm scan protocols. Further, the small sample size may have also introduced heterogeneity in the analysis of mGCC thickness, although sensitivity analyses proved the reliability of our results. Further studies should be included to comprehensively evaluate the influence of scan area and protocols on the assessment of macular structure changes in PPG, EG, and OHT. Moreover, the heterogeneity was high regarding many of our findings, indicating that the results should be cautiously interpreted.

## Conclusion

The OCT-based assessment of peripapillary and macular structural changes could be potentially utilized to discriminate PPG from EG and OHT. This facilitates a better understanding of the pathophysiology of glaucoma and provides references for ophthalmologists to manage individuals suspected to have glaucoma and glaucoma patients according to the extent of severity in a non-invasive way. Further studies that employ clock hour classification methods that can monitor the configuration alterations in a narrower range and longitudinal studies are needed to verify our findings.

## Data Availability Statement

The original contributions presented in the study are included in the article/Supplementary Material, further inquiries can be directed to the corresponding author/s.

## Author Contributions

YT, TW, XZ, and BJ: conceptualization and design. YT, TW, and YH: literature search, data extraction, quality assessment, and data analysis. YT, TW, XZ, YH, and BJ: manuscript writing and editing. BJ: supervision. All authors approved the final version of the manuscript.

## Conflict of Interest

The authors declare that the research was conducted in the absence of any commercial or financial relationships that could be construed as a potential conflict of interest.
