# Supplementary material for: Optical Coherence Tomography Evaluation of Peripapillary and Macular Structure Changes in Pre-perimetric Glaucoma, Early Perimetric Glaucoma, and Ocular Hypertension: A Systematic Review and Meta-Analysis
Source: Front Med (Lausanne). 2021 Jul 1;8:696004. doi: 10.3389/fmed.2021.696004 (PMC8280320; doi:10.3389/fmed.2021.696004)
Supplement: Supplementary file 1 [file Data_Sheet_1.pdf]

Supplementary figures

Figure S1. Subgroup analysis of average pRNFL thickness in patients with PPG and OHT according to the type of OCT.
Figure S2. Funnel plot of average and sectoral pRNFL thickness in patients with PPG and EG. **(A)** Average. **(B)** Superior. **(C)** Nasal. **(D)** Inferior. **(E)** Temporal.
Figure S3. Funnel plot of average and sectoral mGCC thickness in patients with PPG and EG. **(A)** Average. **(B)** Superior. **(C)** Inferior.
Figure S4. Funnel plot of average and sectoral pRNFL thickness in patients with PPG and OHT. **(A)** Average. **(B)** Superior. **(C)** Inferior.
Figure S5. Funnel plot of average and sectoral mGCC thickness in patients with PPG and OHT. **(A)** Average. **(B)** Superior. **(C)** Inferior.
Figure S6. Funnel plot of average and sectoral mGCIPL thickness in patients with PPG and EG. **(A)** Average. **(B)** Superior. **(C)** Inferior.
Figure S7. Sensitivity analysis of pRNFL thickness in patients with PPG and EG. **(A)** Average. **(B)** Superior. **(C)** Nasal. **(D)** Inferior. **(E)** Temporal.
Figure S8. Sensitivity analysis of mGCIPL thickness in patients with PPG and EG. **(A)** Average. **(B)** Superior. **(C)** Inferior.
Figure S9. Sensitivity analysis of mGCC thickness in patients with PPG and EG. **(A)** Average. **(B)** Superior. **(C)** Inferior.
Figure S10. Sensitivity analysis of pRNFL thickness in patients with PPG and OHT. **(A)** Average. **(B)** Superior. **(C)** Inferior.
Figure S11. Sensitivity analysis of mGCC thickness in patients with PPG and OHT. **(A)** Average. **(B)** Superior. **(C)** Inferior.
Figure S12. Average mGCIPL thickness in patients with PPG and EG after excluding the study of Kim et al.
Figure S13. Funnel plot of average GCIPL thickness in patients with PPG and EG after excluding the study of Kim et al.
Figure S14. Average mGCC thickness in patients with PPG and EG after excluding the study of Aydogan et al.
Figure S15. Average mGCC thickness in patients with PPG and OHT after excluding the study of Aydogan et al.

Study or Subgroup	PPG Mean	PPG SD	PPG Total	OHT Mean	OHT SD	OHT Total	Weight	Mean Difference IV, Fixed, 95% CI
**1.1.1 SD-OCT**								
Aydogan 2017	102.5	5.8	94	112.2	6.3	77	50.7%	-9.70 [-11.53, -7.87]
Garas 2011	94.9	12.3	46	102.7	10.7	36	6.8%	-7.80 [-12.79, -2.81]
Hollo 2014	94.4	12.48	33	102.6	8.34	28	6.1%	-8.20 [-13.46, -2.94]
Sazenoz 2020	88.04	8.47	28	92.88	11.06	18	4.7%	-4.84 [-10.84, 1.16]
**Subtotal (95% CI)**			**201**			**159**	**68.4%**	**-9.04 [-10.62, -7.46]**
Heterogeneity: Chi² = 2.72, df = 3 (P = 0.44); I² = 0%								
Test for overall effect: Z = 11.24 (P < 0.00001)								
**1.1.2 TD-OCT**								
Pomorska 2012	84	12.8	27	95.1	7.54	33	5.7%	-11.10 [-16.57, -5.63]
Taliantzis 2009	90.12	5.28	42	96.9	7.52	54	25.9%	-6.78 [-9.34, -4.22]
**Subtotal (95% CI)**			**69**			**87**	**31.6%**	**-7.56 [-9.88, -5.24]**
Heterogeneity: Chi² = 1.96, df = 1 (P = 0.16); I² = 49%								
Test for overall effect: Z = 6.38 (P < 0.00001)								
**Total (95% CI)**			**270**			**246**	**100.0%**	**-8.57 [-9.88, -7.27]**
Heterogeneity: Chi² = 5.75, df = 5 (P = 0.33); I² = 13%								
Test for overall effect: Z = 12.88 (P < 0.00001)								
Test for subgroup differences: Chi² = 1.07, df = 1 (P = 0.30), I² = 6.6%								

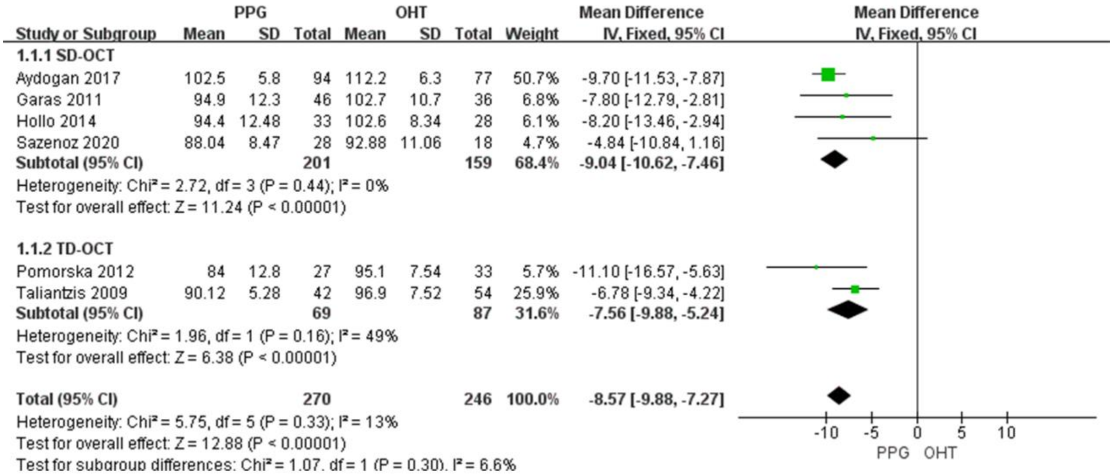

Figure S1. Subgroup analysis of average pRNFL thickness in patients with PPG and OHT according to the type of OCT.

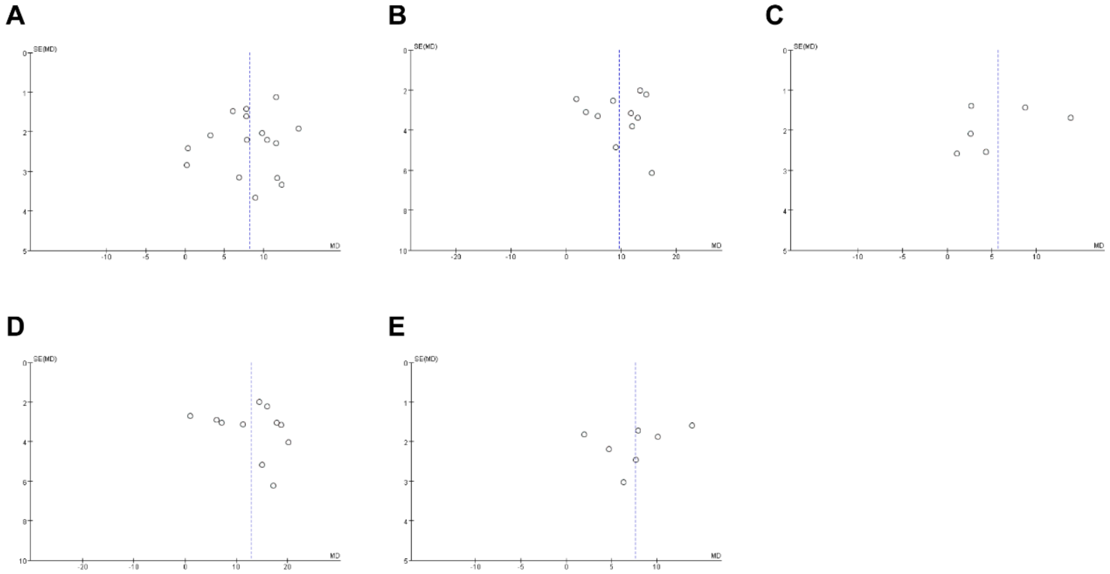

Figure S2. Funnel plot of average and sectoral pRNFL thickness in patients with PPG and EG. **(A)** Average. **(B)** Superior. **(C)** Nasal. **(D)** Inferior. **(E)** Temporal.

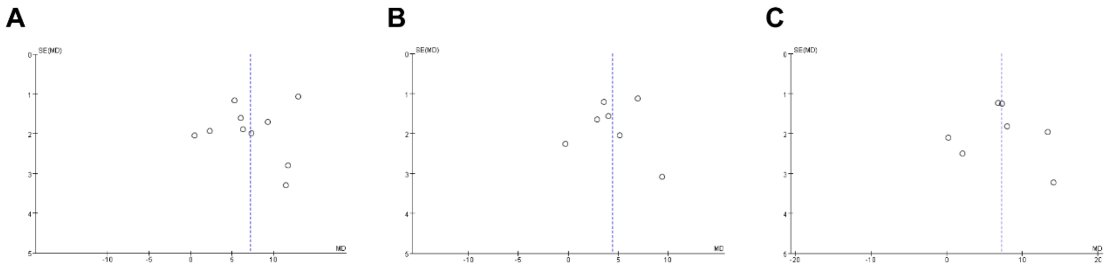

Figure S3. Funnel plot of average and sectoral mGCC thickness in patients with PPG and EG. **(A)** Average. **(B)** Superior. **(C)** Inferior.

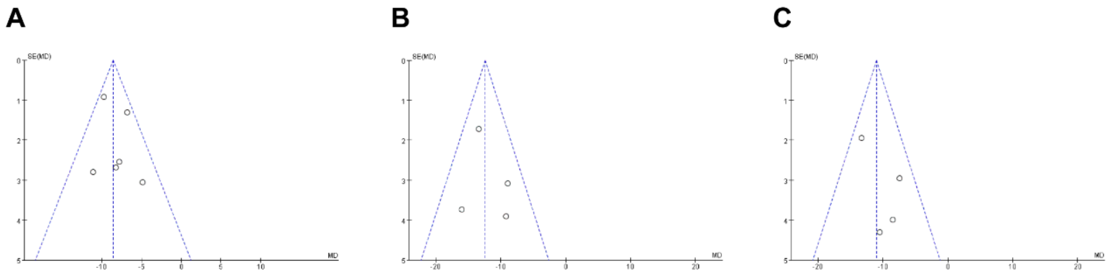

Figure S4. Funnel plot of average and sectoral pRNFL thickness in patients with PPG and OHT. **(A)** Average. **(B)** Superior. **(C)** Inferior.

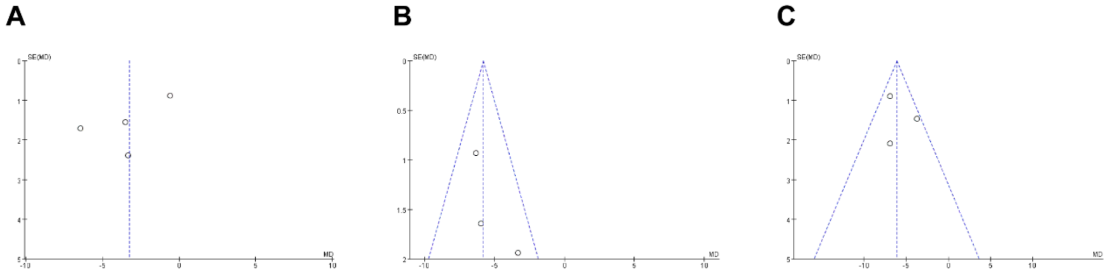

Figure S5. Funnel plot of average and sectoral mGCC thickness in patients with PPG and OHT. **(A)** Average. **(B)** Superior. **(C)** Inferior.

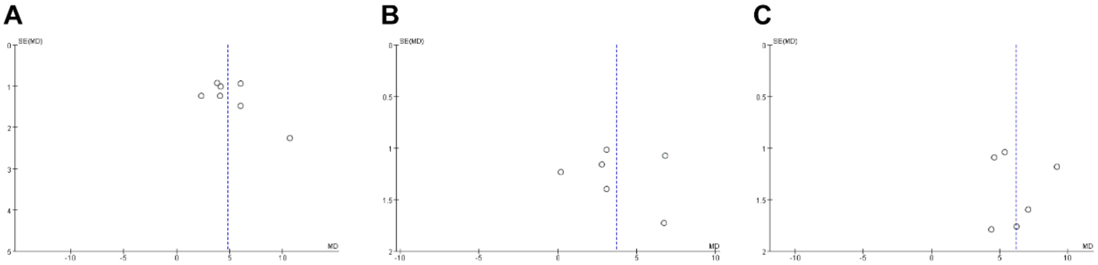

Figure S6. Funnel plot of average and sectoral mGCIPL thickness in patients with PPG and EG. **(A)** Average. **(B)** Superior. **(C)** Inferior.

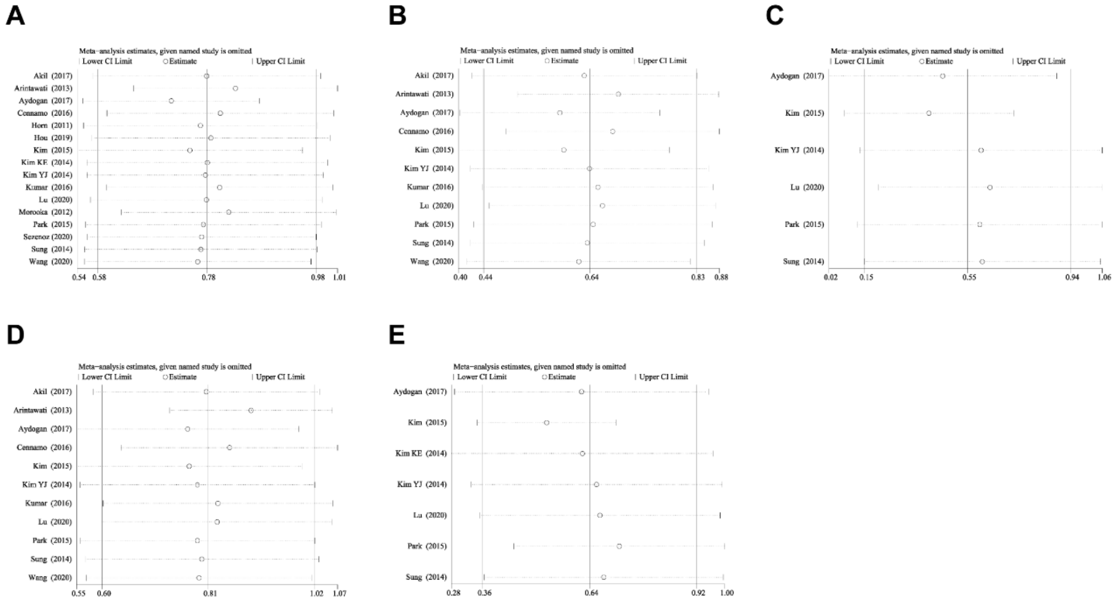

Figure S7. Sensitivity analysis of pRNFL thickness in patients with PPG and EG. **(A)** Average. **(B)** Superior. **(C)** Nasal. **(D)** Inferior. **(E)** Temporal.

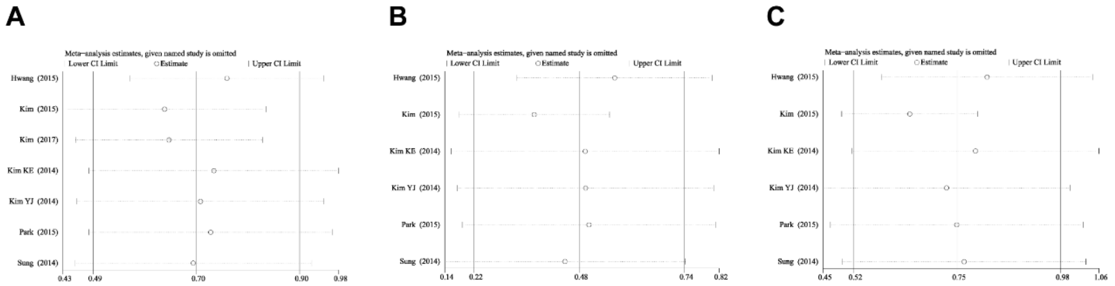

Figure S8. Sensitivity analysis of mGCIPL thickness in patients with PPG and EG. **(A)** Average. **(B)** Superior. **(C)** Inferior.

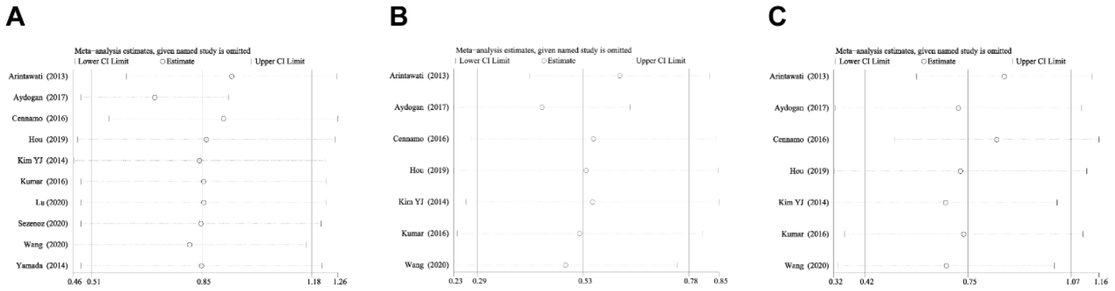

Figure S9. Sensitivity analysis of mGCC thickness in patients with PPG and EG. **(A)** Average. **(B)** Superior. **(C)** Inferior.

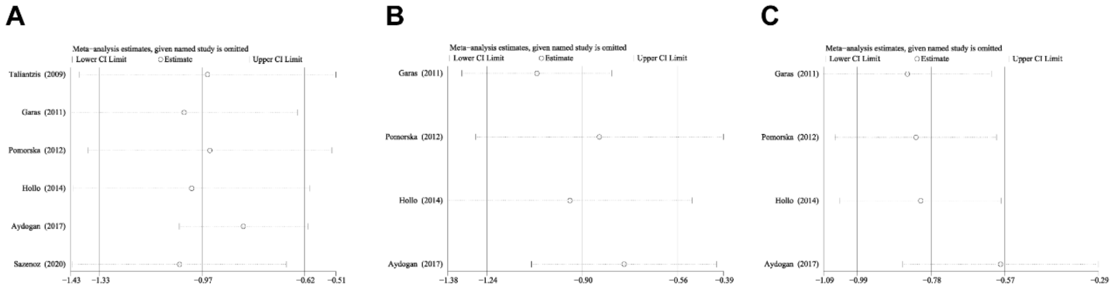

Figure S10. Sensitivity analysis of pRNFL thickness in patients with PPG and OHT. **(A)** Average. **(B)** Superior. **(C)** Inferior.

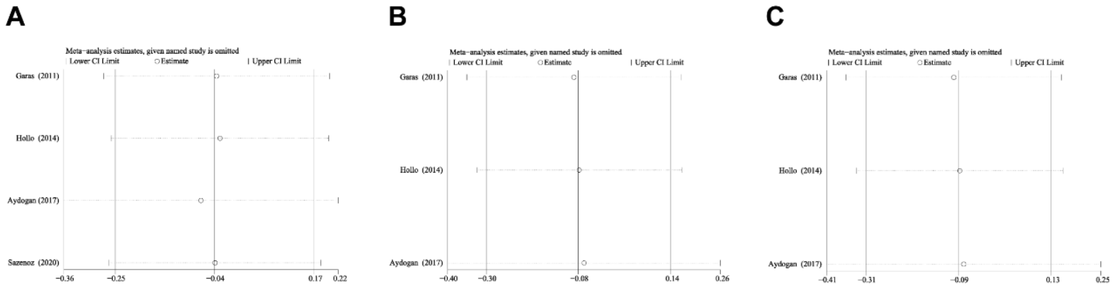

Figure S11. Sensitivity analysis of mGCC thickness in patients with PPG and OHT. **(A)** Average. **(B)** Superior. **(C)** Inferior.

Study or Subgroup	PPG Mean	PPG SD	PPG Total	EG Mean	EG SD	EG Total	Weight	Mean Difference IV, Random, 95% CI
Hwang 2015	72.5	6.9	48	70.2	7.7	110	14.3%	2.30 [-0.13, 4.73]
Kim 2015	78.9	5.5	79	72.9	6.5	83	20.4%	6.00 [4.15, 7.85]
Kim 2017	75.75	7.45	26	65.09	8.82	26	0.0%	10.66 [6.22, 15.10]
Kim KE 2014	77.3	6.6	103	73.5	6.9	111	21.0%	3.80 [1.99, 5.61]
Kim YJ 2014	66.49	5.36	68	62.36	6.55	72	18.8%	4.13 [2.15, 6.11]
Park 2015	76	7.2	50	71.9	7.2	106	14.4%	4.10 [1.68, 6.52]
Sung 2014	78.24	6.55	37	72.21	8.42	70	11.0%	6.03 [3.14, 8.92]
**Total (95% CI)**			**385**			**552**	**100.0%**	**4.39 [3.30, 5.47]**

Heterogeneity: Tau² = 0.61; Chi² = 7.53, df = 5 (P = 0.18); I² = 34%
Test for overall effect: Z = 7.91 (P < 0.00001)

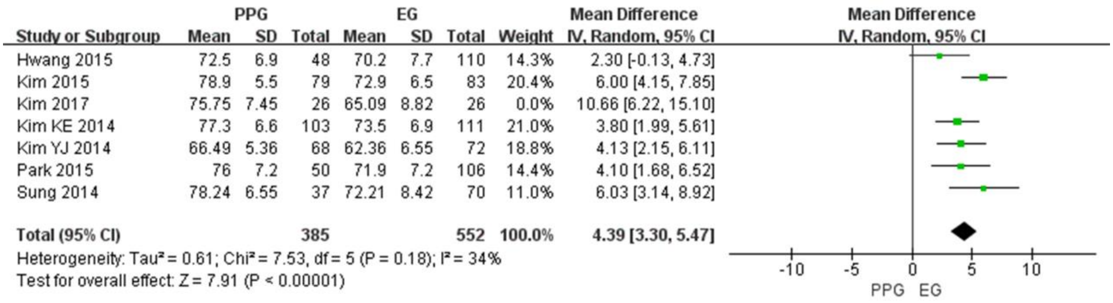

Figure S12. Average mGCIPL thickness in patients with PPG and EG after excluding the study of Kim et al.

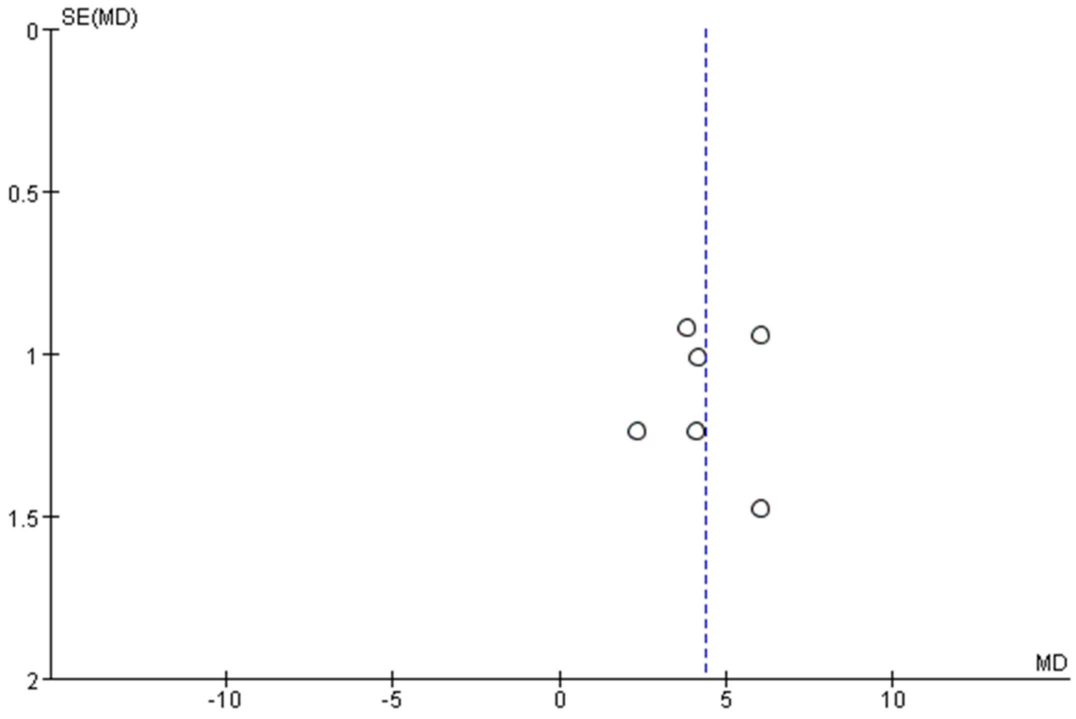

Figure S13. Funnel plot of average GCIPL thickness in patients with PPG and EG after excluding the study of Kim et al.

	PPG			EG				Mean Difference
Study or Subgroup	Mean	SD	Total	Mean	SD	Total	Weight	IV, Random, 95% CI
Arintawati 2013	83.82	9.85	32	83.34	9.59	81	10.9%	0.48 [-3.52, 4.48]
Aydogan 2017	99.9	6.6	94	87	6.7	66	0.0%	12.90 [10.80, 15.00]
Cennamo 2016	92.58	4.99	66	90.27	11.74	41	11.4%	2.31 [-1.48, 6.10]
Hou 2019	98.6	8.65	68	93.3	6.36	162	15.0%	5.30 [3.02, 7.58]
Kim YJ 2014	100.53	9.17	68	91.24	10.98	72	12.4%	9.29 [5.95, 12.63]
Kumar 2016	90.9	7.35	28	84.9	7.47	83	12.9%	6.00 [2.84, 9.16]
Lu 2020	90.2	7.22	44	82.86	10.76	42	11.2%	7.34 [3.45, 11.23]
Sezenoz 2020	104.33	8.48	28	92.92	16.04	31	6.6%	11.41 [4.95, 17.87]
Wang 2020	92.4	7.9	26	80.7	10.9	22	8.1%	11.70 [6.23, 17.17]
Yamada 2014	96.1	8.7	30	89.8	5.7	31	11.6%	6.30 [2.60, 10.00]
**Total (95% CI)**			**390**			**565**	**100.0%**	**6.28 [4.20, 8.37]**

Heterogeneity: Tau² = 6.19; Chi² = 22.37, df = 8 (P = 0.004); I² = 64%

Test for overall effect: Z = 5.91 (P < 0.00001)

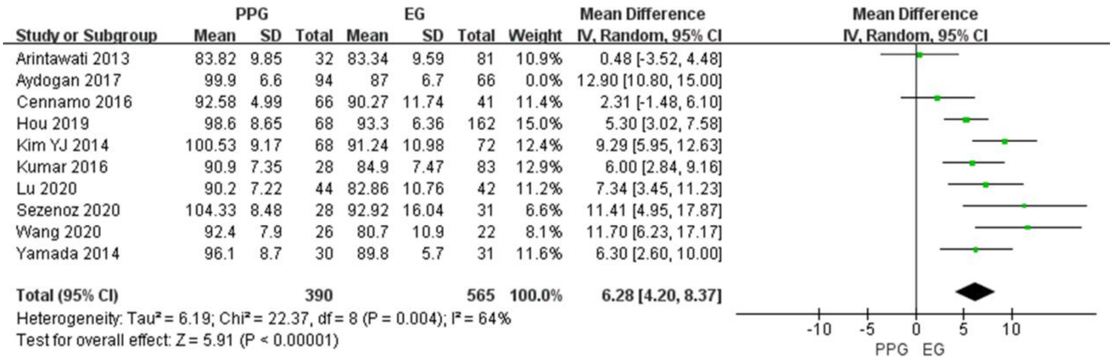

Figure S14. Average mGCC thickness in patients with PPG and EG after excluding the study of Aydogan et al.

Study or Subgroup	PPG Mean	PPG SD	PPG Total	OHT Mean	OHT SD	OHT Total	Weight	Mean Difference IV, Fixed, 95% CI
Aydogan 2017	99.9	6.6	94	100.5	4.9	77	0.0%	-0.60 [-2.33, 1.13]
Garas 2011	91.8	7	46	95.3	7	36	44.4%	-3.50 [-6.55, -0.45]
Hollo 2014	91.12	7.08	33	97.55	6.29	28	36.7%	-6.43 [-9.79, -3.07]
Sazenoz 2020	104.33	8.48	28	107.64	7.49	18	18.9%	-3.31 [-7.98, 1.36]
**Total (95% CI)**			**107**			**82**	**100.0%**	**-4.54 [-6.57, -2.51]**

Heterogeneity: Chi² = 1.93, df = 2 (P = 0.38); I² = 0%

Test for overall effect: Z = 4.38 (P < 0.0001)

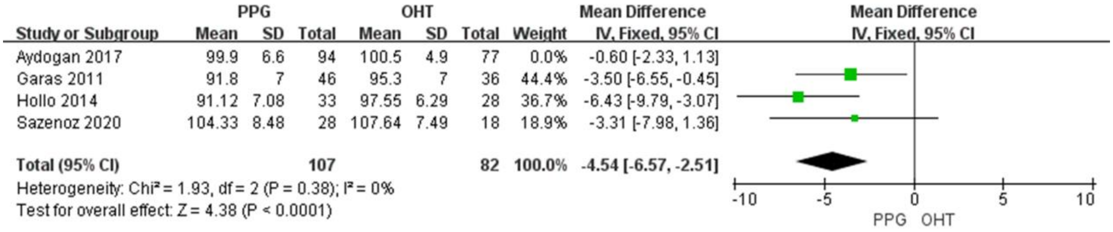

Figure S15. Average mGCC thickness in patients with PPG and OHT after excluding the study of Aydogan et al.
